# Double-Stranded RNA Viral Infection in Tehran *Trichomonas vaginalis* Isolates

**Published:** 2013

**Authors:** S Heidary, M Bandehpour, Z Valadkhani, SJ Seyyed–Tabaee, A Haghighi, AR Abadi, B Kazemi

**Affiliations:** 1 Dept. of Parasitology and Mycology, Shahid Beheshti University of Medical Sciences, Tehran, Iran; 2 Cellular and Molecular Biology Research Center, Shahid Beheshti University of Medical Sciences, Tehran, Iran; 3 Dept. of Biotechnology, Shahid Beheshti University of Medical Sciences, Tehran, Iran; 4 Dept. of Parasitology, Pasteur Institute of Iran, Tehran, Iran; 5 Dept. of Social Medicine, Shahid Beheshti University of Medical Sciences, Tehran, Iran

**Keywords:** *Trichomonas vaginalis*, Viral infection, dsRNA virus

## Abstract

**Background:**

*Trichomonas vaginalis* is a pathogenic protozoon and may be contaminated with dsRNA virus called *Trichomonas vaginalis virus* (TVV). The viral infection is an important factor for its pathogenesis and sensitivity to metronidazole. The presence of TVV is associated with qualitative and quantitative expression of cysteine proteinases and surface immunogenic; P270. The purpose of this study was to determine TVV frequency in *T. vaginalis* clinical isolates in Tehran, Iran.

**Methods:**

The 46 *T. vaginalis* isolates were collected from Tehran Province and cultured in TYI-S-33 culture medium. Viral RNA was extracted and RT-PCR was done.

**Results:**

Of 46 *T. vaginalis* isolates, 8 isolates (17.39%) were infected with TVV-1. There was not any association between patient age and TVV- infected *T. vaginalis*. There were 17.39% viral infection in *T. vaginalis* isolates which was lower than that reported by other researchers.

**Conclusion:**

This is the first report on *T. vaginalis* isolates infection by TVV-1 in Iran.

## Introduction

*Trichomonas vaginalis* is a parasitic protozoa causes trichomoniasis, a disease of global importance (1). *Trichomonas vaginalis* infection increases risk of HIV (2), premature labor during pregnancy (3), cervical cancer (4), pelvic inflammatory disease (5) and infertility(6). Some *T. vaginalis* isolates are infected by a double-stranded RNA virus called *T. vaginalis* virus (TVV), a divergent group of Totiviridae virus family. It contains three viral genotypes and three satellite dsRNA species. The existence of various dsRNA virus species in some *T.vaginalis* isolates defines the presence of mixed viral infection. The vertical transmission of TVV occurs in cellular mitotic division time (7).

TVV is described as a heterogeneous population of icosahedral, filamentous, cylindrical and spherical viral particles with a size of 33-200 nm (8, 9). The genome size of the dsRNA is variable as 4.3 to 5.0 kb (10). Electron microscopy studies show that *T. vaginalis* isolates can be simultaneously infected by several types of virus-like particles (VLPs) (11). This viral infection may be an important aspect for *T. viginalis* virulence and pathogenesis; on the other hand, it is also seen in the metronidazole sensitive strains of *T. viginalis*. TVV increases the surface expression of the immunogenic protein called P270 (12).

The presence and replication of the viral dsRNA causes the phenotypic changes for the P270 prominent immunogenic among infected trichomonads. There is a relationship between the presence of TVV and upregulation of a major cellular immunogenic (12).

Phosphorylation of P270 and cytoplasmic expression occurs in high iron rate. The iron level in medium is associated with the P270 surface expression in dsRNA- infected *T. vaginalises*. Furthermore, the P270 motion among cytoplasmic and plasma membrane which is iron-regulated is only observed in TVV infected *T.vaginalis* (13).

The difference in p270 gene expression in multiple virus infected *T. vaginalis* can make various genetic backgrounds in this parasite. The viral proteins may have important role in p270 gene transcription regulation in *T. vaginalis* (14).

TVV has a role in cysteine proteinase expression and quantitative and qualitative changes in the composition of total protein in the parasite (15). In addition, the TVV is responsible for different phenotypic changes in *T. viginalis* that may have impact on virulence of parasite (16).

It is hypothesised that TVV can be able to translate the reductase enzyme which is responsible for reducing metronidazole and consequently drug sensivity (8).

The aim of this study was to determine TVV frequency in *T. vaginalis* clinical isolates in Tehran.

## Materials and Methods

### Collection and culture of Trichomonas viginalis isolates

Forty-six isolates of *T. viginalis* of vaginal discharge and urine samples were collected from Tehran Province, Iran during 2011-2012. The vaginal secretions and urine samples were cultured on the TYI-S-33 Diamond's medium (17), supplemented with 10% heat-inactivated bovine calf serum at 37 °C. Antibiotics (100 U / mL penicillin, 30 µg / mL streptomycin sulfate) and anti-fungus (40µg/mL amphotericin B) were added to culture medium. Parasites were washed and concentrated in exponential phase at 2×10^6^ cells per sample and saved at the – 80 °C for further work.

This study was approved by university ethics committee. An informed consent was written for each participant.

### RNA Extraction

Samples were washed by 1x PBS (pH 7.4) for one time using centrifugation for 10 min at 4 °C at 8000×g. The nucleic acids were extracted using GeneJET^TM^ RNA purification kit (K0731, Fermentas) according to manufacturer's instructions.

### cDNA Synthesis

Five micrograms of viral dsRNA were heated for 5 min at 95 °C, and used as template for cDNA synthesis. The reaction mixture contained 200 units of RevertAid Reverse Transcriptase (Fermentas), 1x reaction buffer ( 5x reaction buffer contain: 50 mM Tris HCl pH 8.3, 50 mM KCl, 4 mM MgCl2, 10 mM dithiothreitol), 0.2 mM dNTPs, and 20 pmol each of forward and reverse primer in 20 microliter final volume. Reaction was placed for 60 min at 42 °C. For inactivation of Reverse transcriptase enzyme, reaction was placed at 95°C for 5 min.

A pair of primers was designed based on TVV1 isolate Changchun capsid protein gene (Accession DQ528812.1).

TVV1 F: 5′- CAC GCA CAT CTC AGA CAG TC -3′ and TVV1 R: 5′- GGG ATG GTT CCT GTA GTT C -3′.

### PCR amplification

The PCR reaction was contained 20 pmol each of forward and reverse primer, 0.2 mM dNTPs, 1.5 unit of Taq DNA polymerase enzyme, 1.5 mM MgCl2, and 1x PCR buffer in 20 micro liters final volume. The reaction was done with following conditions for 40 cycles: denaturation at 94°C for 30 seconds, primer annealing at 51°C for one minute and extension at 72 °C for 30 seconds. PCR products were electrophoresed on 2% agarose gel. The primers were amplified 204bp of TVV Capsid gene.

## Results

### Agarose gel electrophoresis

**Fig. 1:**
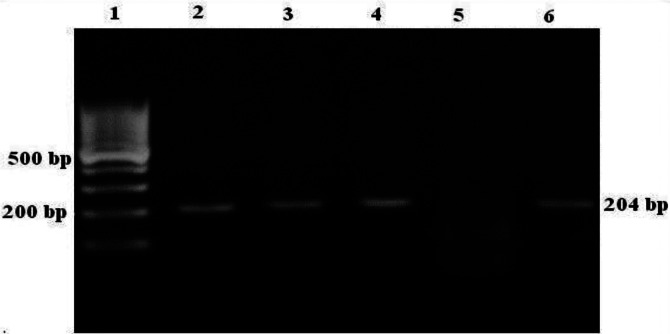
2% agarose gel electrophoresis of PCR products. Lane 1: 100 bp DNA ladder marker. Lanes 2, 3, 4 and 6: virus positive samples, lane 5: virus negative sample

*Trichomonas vaginalis* isolates were investigated using RT-PCR for TVV type 1. The 8 (17.39%) isolates of 46 were infected with TVV-1, and a 204 bp band was demonstrated on agarose gel electrophoresis (Fig. 1). PCR products were purified, sequenced and deposited to GenBank at accession numbers: AB701559, AB701560, AB701561, AB701562, AB701563, AB701564, AB701565, and AB701566.

### Statistical analysis

All informations were analyzed using SPSS16 software, the Mann-Whitney statistical analysis showed no significant relationship between age and the presence of TVV in *T. vaginalis*.

## Discussion

Since the presence of TVV in *T.vaginalis* is related to parasite virulence and pathogenesis, moreover, the TVV cause the increase metronidazole sensitivity, thus, the virus frequency in *T.vaginalis* clinical isolates is healthy important

Double-stranded RNA was not seen in vertebrates, but pieces of double-stranded RNA have been observed in phage-infected fungi (18), rat liver cells (19) and Burkitt lymphoma cells (20). Wang et al. reported double-stranded RNA virus in *Giardia* (21) and *T. vaginalis* (8). TVV is described as heterogeneous populations of icosahedral, filamentous, cylindrical, and/or spherical virus particles with a size of 33-200 nm (8, 9). The parts of the dsRNA genome size vary from 4.3 kbp to 5 kbp (10). It is believed that the virus is found in strains sensitive to metronidazole (22). The 17.39% infection rate of *T. vaginalis virus* type 1 reported in this study is lower than that of reported by others. Snipes et al. reported 50% infection rate in 109 *T. vaginalis* isolates in the USA (22). Hampel et al. reported the infection rate of 44% when analyzed 20 isolates from different geographic origins (23) and also Fraga et al. reported 55% infection rate in 40 *T. vaginalis* isolates from Havana, Cuba (24). The high infection rates of 81.9% and 75% have been reported in 72 isolates in South Africa (25) and 28 isolates in the USA, respectively (26). On the other hand, the 17.39% infection rate reported in this study is more than of 14% infection rate reported in 22 isolates from Korea (27).

It seems that the difference in TVV infection rate in various studies is because of the difference in geographical zones and may be due to the difference in the method for virus detection and sample size. We used RT PCR for virus detection but others used electrophoresis of virus RNA genome.

A significant relationship between patients age and the presence of TVV in *T. vaginalis* has been reported. The patients infected with positive virus isolates are older than ones infected with negative virus isolates (26). However in this study there was no relationship between patients age and TVV presence. Our sample size is more than Wendel et al. (26), but results are different (17.39% and 75% respectively). So it can be said that examination on the more samples due to the better statistical conclusion.

## Conclusion

This is the first report on *T. vaginalis* isolates infection by TVV-1 in Iran. There was 17.39% infection frequency rate in *T. vaginalis* isolates by dsRNA virus.
